# Assessment of DNA Repair Gene Expressions in Vitrified Mouse Preantral Follicles

**DOI:** 10.22074/cellj.2020.6865

**Published:** 2020-09-08

**Authors:** Zahra Khodavandpour, Saeed Zavareh, Parisa Farrokh, Meysam Nasiri

**Affiliations:** 1 Department of Biology, Damghan Branch, Islamic Azad University, Damghan, Iran; 2 School of Biology, Damghan University, Damghan, Iran; 3 Institute of Biological Sciences, Damghan University, Damghan, Iran

**Keywords:** DNA Repair, Ovarian Follicles, Vitrification

## Abstract

**Objective:**

Vitrification of the ovarian tissue is one of the techniques recommended for preserving the fertility of women who are dealing with infertility. Despite its benefits, our information about the molecular aspects of ovarian follicles vitrification is somehow ambiguous. Therefore, the aim of this study was to evaluate the expression pattern of DNA repair genes in vitrified preantral follicles.

**Materials and Methods:**

In this experimental study, the isolated preantral follicles (n=906) from 14-16 days old mice (n=12) were divided into three groups: fresh, toxic and vitrified which were cultured in vitro for 12 days. Preantral follicles were vitrified using cryotop followed by exposure to equilibration solution for five minutes and vitrification solution (VS) for 30 seconds. In the toxic group, preantral follicles were only placed in equilibration and vitrification media and they were then placed in the warming solutions without exposure to liquid nitrogen. On the second and sixth days of the culture period, real-time quantitative reverse transcription-polymerase chain reaction (qRT-PCR) was carried out to evaluate expression of the selected genes involved in DNA repair, including Msh6 (MutS homolog 6), Mre11 (Meiotic recombination 11), Brca1 (Breast cancer type 1), Rad51 (RAD51 recombinase), Pcna (Proliferating cell nuclear antigen) and Atm (ATM serine/threonine kinase). In addition, developmental parameters including growth, survival rate, antrum cavity formation and ovulation were analyzed.

**Results:**

The relative mRNA expression of Msh6, Mre11, Brca1, Rad51, Pcna and Atm on the second and sixth days of the culture period in vitrified group was significantly higher than those of the control and toxic groups, but there was no significant difference between the toxic and control groups. In addition, developmental parameters of follicles were similar in both toxic and control groups, while both were significantly higher than that of vitrified group.

**Conclusion:**

Vitrification changes the expression pattern of DNA repair genes of the mouse preantral follicles.

## Introduction

Not only it is vital to survive the patient who are dealing with cancer, but also increase the quality of life and patient satisfaction following treatment is important (1). The cytotoxic effect of radiation and chemical factors in cancer often results in premature ovarian failure (2). In this regard, assisted reproduction techniques such as vitrification of ovarian tissue, follicle, oocyte and embryo can preserve the fertility. Vitrification methods allow longterm storage of any types of cells. However, vitrification may have negative effects on the molecular level, such as DNA, mRNA and proteins, which their manifestations are not immediately expressed after thawing and sometimes does not cause death (3). It has been proposed that vitrification leads to DNA damage and results in damage to cytoplasmic mRNA (4). However, there are some debated results about the effects of vitrification on gene expression levels of ovarian follicles (5, 6). But, there are not any reports about the effect of vitrification on DNA repair genes of ovarian follicles. However, In this regard Fatehi et al. (7) have showed the vitrification of follicles did not change gene expression pattern related to folliculogenesis such as *Bmp15, Gdf9, BmprII, Alk6, Alk5, Has2,* and *Ptgs*. Additionally, Sampaio da Silva et al. (8) have recently reported that vitrification decreased proliferation of granulosa cells in developing follicles via changing the expression of *Cx43* gene. Moreover, Asadzadeh et al. (9) showed that expression of gelatinase related genes altered in follicles isolated from vitrified ovarian tissue.

Stressors, either internal or external, which lead to DNA damages may result in activation of DNA repair systems (10, 11). One of the DNA repair pathways is DNA mismatch repair (MMR), activated during 99% of the DNA damages. PCNA and MSH6 proteins play key roles in identifying and repairing DNA mismatches (11). In addition, PCNA plays an important role in many essential cellular processes, such as DNA replication, cell cycle control, cell survival and regulation of ovarian follicles development (10). Another pathway in DNA repair is homologous recombination (*HR*). In this pathway, ATM protein, along with other factors, plays a key role in the binding of broken DNA strands. Previous studies have demonstrated that ATM protein deficiency may result in oocyte apoptosis at the prophase stage and female infertility (10-12). Any failure in the doublestranded DNA structure activate ATM protein which interacts with RAD51C/BRCA2 and form a complex to repair DNA, using an intact strand as a template (10-12). Formation of the Mre11a3, Rad50 and Nsb1 complexes is essential for HR repair pathway (10-13) and any defection in this complex causes cell apoptosis. Furthermore, the interaction of BRCA, FANC1/FANC2 and RAD 51 is also essential to complete HR repair pathway. Thus, any mutation in *Brca* genes are associated with the increasing of breast and ovarian cancers (10, 12). The *BRCA1* and *BRCA2* tumor suppressor genes prevent tumor development through repair of DNA damage. Any mutation in aforementioned genes result in malignancy (14). It was demonstrated that BRCA1, in collaboration with RAD51 has a key role in HR repair pathway (10-12).

Regarding to the importance of DNA repair process in the control of cell growth and developmental competence in ovarian follicles (3, 12), the aim of this study was to investigate effect of vitrification of mice preantral follicles on the DNA repair gene expressions.

## Materials and Methods

All chemicals were purchased from Sigma-Aldrich (UK) Company, otherwise those mentioned in the text, as well as culture media made with the Deionized water (Milli-Q).

### Animals

In this experimental study, NMRI mice (14-16 days old, n=12) were obtained from the Razi Vaccine and Serum Research Institute of Iran and they were kept in appropriate conditions including: 12 hours of darkness and 12 hours of light, temperature of 20-24°C and 40- 50% of moisture with free access to the food and water. Experiments of the present study has been reviewed and approved by Ethical Committee of the School of Biology, Damghan University (IR. BSDU.REC.1397.14). All applicable institutional guidelines for the care and use of animals were followed in accordance with declaration of Helsinki as revised in Tokyo 2004.

### Isolation of preantral follicles

The mice were euthanized through cervical dislocation and the ovaries were removed and placed in 200 μl drops of α-MEM medium containing 10% fetal bovine serum (FBS, Sigma-Aldrich, Australia), 2.2 g/l sodium bicarbonate, 100 IU/ml penicillin, 75 μg/ml streptomycin and 25 mM HEPES. Preantral follicles were isolated mechanically as described previously (15) using a G29 needle connected to an insulin syringe under a stereomicroscope (Olympus, Japan) ×25 magnification. After that, preantral follicles with an approximate diameter of 140-160 μm containing a central oocyte and 2-3 layers of granulosa cells were considered healthy and selected for culture *in vitro*.

### Experimental design

The preantral follicles were randomly divided into three groups (1). Control group: fresh preantral follicles that were cultured for 12 days, immediately after isolation (2). Vitrification group: vitrified preantral follicles thawed and cultured for 12 days (3). Toxic group: preantral follicles without exposure to liquid nitrogen were only placed in equilibration and vitrification media and they were cultured for 12 days. The experiment was conducted in two steps. First, the growth and development of preantral follicles were evaluated and then DNA repair gene expressions were assessed separately.

### Vitrification of preantral follicles

The vitrification of preantral follicles were carried out as described previously by Hatami et al. (16) with slightly modifications. The preantral follicles were kept in an equilibrium solution (ES) composed of phosphate-buffered saline (PBS) supplemented with ethylene glycol (EG, 7.5% V/V), dimethyl sulfoxide (DMSO, 7.5% V/V) and FBS (20%) for 5 minutes. After that, the preantral follicles were transferred to vitrification solution (VS) composed of PBS supplemented with EG (15% V/V), DMSO (15% V/V), FBS (20%) and sucrose (0.5 M) and they were kept for 30 seconds. Subsequently, preantral follicles were immediately removed from VS using a Pasteur pipette and loaded to the thin end of the cryotop tape and transferred to the liquid nitrogen. For warming, the preantral follicles were kept in PBS solution supplemented with a descending concentration of sucrose (1, 0.5, 0.25 and 0.125 M sucrose) at an interval of 5 minutes. In the toxic group: preantral follicles were only placed in equilibration and vitrification media and then without exposure to liquid nitrogen placed in the warming solutions (descending concentration of sucrose: 1, 0.5, 0.25 and, 0.125 M sucrose at an interval of 5 minutes) to remove cryoprotectant.

### *In vitro* culture of preantral follicles

Preantral follicles were cultured in 25 μl drops of α-MEM culture medium enriched with 5% FBS, 0.1 IU/ml human follicle stimulating hormone (hFSH), 1% insulin, transferring and selenium (ITS), 10 ng/ml of epidermal growth factor (EGF), 2.2 g/l sodium bicarbonate, 100 IU/ ml penicillin and 75 μg/ ml streptomycin under mineral oil in incubator at 37°C, 90% humidity and 5% CO_2_ for 12 days. Every 48 hours, approximately 15 μl of the culture medium from each drop was replaced by a fresh medium.

During the culture period, diameter and morphological changes were evaluated. Follicle diameter was measured by an inverted microscope (Nikon, Japan) under ×400 magnification through calculating the average of two perpendicular diameters with a precalibrated ocular micrometer at the initial time (day 0), in addition to the second and fourth days of the culture period. Darkness of follicles and follicles without an oocyte or naked oocyte were considered as degenerated follicles. The antral cavity was defined as a lucent area among the granulosa cells. Survival rate was calculated by the ratio of survived follicles to the total follicles.

### Induction of ovulation

In order to induce ovulation, on the tenth day of the culture period, 1.5 IU/ml of human chorionic gonadotropin (hCG, Choriomon, Switzerland) was added to the culture medium. After 16-48 hours, ovulation rate was considered under invert microscope (Nikon, Japan) and the released oocytes were categorized into three groups: germinal vesicle (GV), metaphase I (MI) and metaphase II (MII).

### RNA extraction

RNA extraction was performed using Trizol^®^ (*Qiagen, Germany*) according to the manufacture’s protocol. In brief, 100 preantral follicles were homogenized in 100 μl of Trizol solution, with vigorously vortex for 3 minutes, followed by incubation at room temperature for 5 minutes. After that 50 μl chloroform was added, gently mixed and incubated at room temperature for 3 minutes. It was then centrifuged in 12000 g for 15 minutes at 4˚C. The upper aqueous phase containing total RNA was carefully removed and placed in a new centrifuge tube. 125μl of Isopropyl alcohol was added and incubates at room temperature for 3 minutes, followed by centrifugation in 12,000 g for 30 minutes at 4˚C. The resultant pellet was washed with 1 ml of 75% ethanol and centrifuged in 7500 g for 5 minutes at 4˚C. The resultant pellet dried at RT and solved in 20 μl sterile water. The integrity and purity of extracted RNA was evaluated by density ratio of 28S to 18S rRNA bands on the agarose gel electrophoresis and measuring the absorbance ratio of 280/260 nm using spectrophotometer, respectively. Samples with 260/280 nm ratio of 1.8-2.0 were acceptable and used for reverse transcription.

### cDNA synthesis and quantitative reverse transcriptionpolymerase chain reaction

Synthesis of cDNA from 500 ng of total RNA was performed using Takara kit (Takara, Japan) according to the manufacturer’s instructions.

Quantitative reverse transcription polymerase chain reaction (qRT-PCR) was carried out to assess relative mRNA expressions of *Msh6, Mre11, Brca1, Rad51, Pcna* and *Atm* genes in preantral follicles on the second and sixth days of culture period. The primer sets were designed to include the introns or span exon/exon junction to avoid residual genomic DNA amplification using the Allele ID software version 7.8 (Premier Biosoft Int, USA, Table 1). All primer pairs were tested to be specific for the target genes. Amplification of no genomic DNA was confirmed using PCR on the none-reverse transcribed RNA as template. Additionally, cDNA of each gene was used as a positive control in a separate microtube in PCR reaction. qRT-PCR was carried out on the Rotor-gen 6000 machine (Corbett Life Science, USA) using the SYBR green (Takara, Japan). The reactions mixture included SYBR green (5 μl), forward primer (0.5 μl), reverse primer (0.5 μl) and cDNA (4 μl) in 10 μl final volume. PCR reactions were set as 95°C for 30 seconds, followed by 40 cycles of denaturation at 95°C for 10 seconds and annealing/extension at 60°C for 45 seconds. The *Pfaffl* method was applied to calculate relative mRNA expressions. Relative expression level of the target genes were normalized to *Ef1*. The specificity of qRT-PCR was assessed by analysis of melting curve and a no-template negative control sample for each gene.

**Table 1:** Oligonucleotide primer sequences for quantitative reverse transcription-polymerase chain reaction


Gene	Primer sequence (5ˊ-3ˊ)	Nucleotides	Product size	Melting temperature	Accession number in NCBI

Msh6	F: AGGAGACAGAGGTGCATGAG	20	135	61.7	NM_010830.2
	R: CAGTTCTTCGCTGCCCAGT	20		62.7	
Mre11	F: ACTTTGAATCAGATGAGGATG	22	119	55.8	NM_001310728.1
	R: CGTACTACGTCCTTCTTTAGT	22		59.4	
Brca1	F: CTGTCTACATTGAACTAGACTC	22	125	51.1	NM_009764.3
	R: GACCTCTACTTCCGTTCGAC	20		53.8	
Rad51	F: CTCTGGCAGCGATGTCCTAG	20	121	55.9	NM_011234.4
	R: AGGTCCATACGTGACGAATAA	22		50.5	
PCNA	F: GTCTCACGTCTCCTTGGTAC	20	113	53.8	NM_011045.2
	R: CTTGGAGTGGTCGTACAGG	20		53.2	
ATM	F: CAGCCTGTAAACCTTCTAGTC	21	152	52.4	NM_007499.2
	R: CTCAGTTATTACTCCACCGAG	21		52.4	
Ef1	F: AGTCGCCTTGGACGTTCTT	19	124	51.1	NM_010106
	R: GTGTAGTTGTAGCATTAGCC	23		55.3	


### Statistical analysis

SPSS software (version 24, IBM, USA) was used for analyzing all data. The Shapiro-Wilk test was used to test the normality of data. If the Sig. value of the Shapiro-Wilk Test is greater than 0.05, the data is normal. The statistical significance of differences in the relative gene expression levels was evaluated by one way analysis of variance (ANOVA) test and post hoc Dunnet test, among the groups. One way ANOVA and post hoc tukey’s tests were applied for assess significant differences in growth and developmental parameters among the groups. Data are presented as mean ± standard deviation of the mean (SD). A P<0.05 was considered statistically significant. Experiments were repeated at least four times.

## Results

### Growth and development of preantral follicles

At the initial time of culture, follicles with similar diameter (140-160 μm) were selected and analyzed. No significant difference was detected among the all groups. On the second day, the diameter of follicles was increased. The follicles attached to the bottom of plate and immobilized due to proliferation of granulosa cells. On the second day, diameter of the preantral follicles of the vitrified group was significantly lower in comparison with the groups of fresh and toxic follicles, while there was no significant difference between toxic and fresh groups (Table 2). On the fourth day of culture, the growth rate increased. So that the shape of follicles became irregular and measuring diameter was impossible. From the sixth day onwards, some spaces were observed among the granulosa cells, which were considered as antrum cavities. In addition, on the fourth day of culturing period, diameter of preantral follicle in the vitrified group was significantly less than those of the control and toxic groups. However, there was no significant difference between follicle diameters of the control and toxicity groups (Table 2). Furthermore, the percentage of degeneration of vitrified follicles (22.5%) was significantly higher than that of follicles of fresh (14.5%) and toxic (14.5%) groups. However, there was no significant difference between degeneration of follicles in the control and toxic group. The rate of antrum cavity was 65.25% in the vitrified group, which was significantly lower than that of control (82.5%) and toxic groups (81.5%, P<0.05). However, there was no significant difference between the rate of antrum cavity formation of the control and the toxic groups. After induction of ovulation, although there was no significant difference between the ovulation rate in the follicles of fresh (78.25%) and the toxic (74.25%) groups, ovulation rate of follicles in both groups were significantly higher than vitrified group (64.75%). As shown in the Table 3, the rate of GV oocytes obtained from preantral follicles in the vitrified group (19.00%) was significantly higher than those of obtained from preantral follicles in the control (13.00%) and the toxic (13.75%) groups (P<0.05). Rate of the germinal vesicle breakdown (GVBD) oocytes in the vitrified group was significantly lower compared to those of control and toxic groups (P<0.05). However, there was no significant difference in the rate of GVBD oocytes between the control and toxic groups. Percentage of MII oocytes obtained from the preantral follicles of vitrified group (29.75) was significantly lower than that of control (46.00) and toxicity (43.50%) groups. While, there was no significant difference between the rates of MII oocyte in the control and the toxicity groups (Table 3).

**Table 2:** Diameter of the preantral follicles during the culture period


Groups	Number of follicles	Follicle diameters		
	n	Mean (µm ± SD)		
		Initial time	2^nd^ day	4^th^ day

Control	300	146.36 ± 6.31	196.92 ± 8.69	294.56 ± 12.36
Toxicity	296	146.00 ± 5.92	202.18 ± 9.40	302.13 ± 8.65
Vitrification	310	146.56 ± 6.05	184.99 ± 14.77^*^	238.02 ± 34.34^*^


In all cases, at least four experimental replicates were performed. *; Indicate significant difference within the groups (P<0.05).

**Table 3:** Maturation rates of cultured preantral follicles


Groups	Number of follicles	Survived	Antrum formation	Ovulated follicles	Stages of oocyte development		
					GV oocytes	MI oocytes	MII oocytes

Control (fresh follicles)	300	256(85.50 ± 1.73)	248(82.50 ± 1.73)	234(78.25 ± 2.50)	40(13.00 ± 1.83)	56(18.75 ± 1.26	138(46.00 ± 1.41)
Toxicity follicles	296	252(86.00 ± 2.16)	229(81.50 ± 5.92)	220(74.25 ± 2.99)	37(13.75 ± 1.89)	55(18.75 ± 3.10	128(43.50 ± 4.43)
Vitrified follicles	310	239^*^(77.25 ± 1.71)	202^*^(65.25 ± 2.63)	200^*^(64.75 ± 1.71)	58^*^(19.00 ± 0.82)	50^*^(16.00 ± 0.82)	92^*^(29.75 ± 1.50)


In all cases, at least four experimental replicates were performed. Data are presented as n (% ± SD). *; Indicates significant difference within the groups (P<0.05), GV; Germinal vesicle oocyte, MI; Metaphase I oocyte, and MII; Metaphase II oocyte.

### Expression of genes

**Fig.1:**
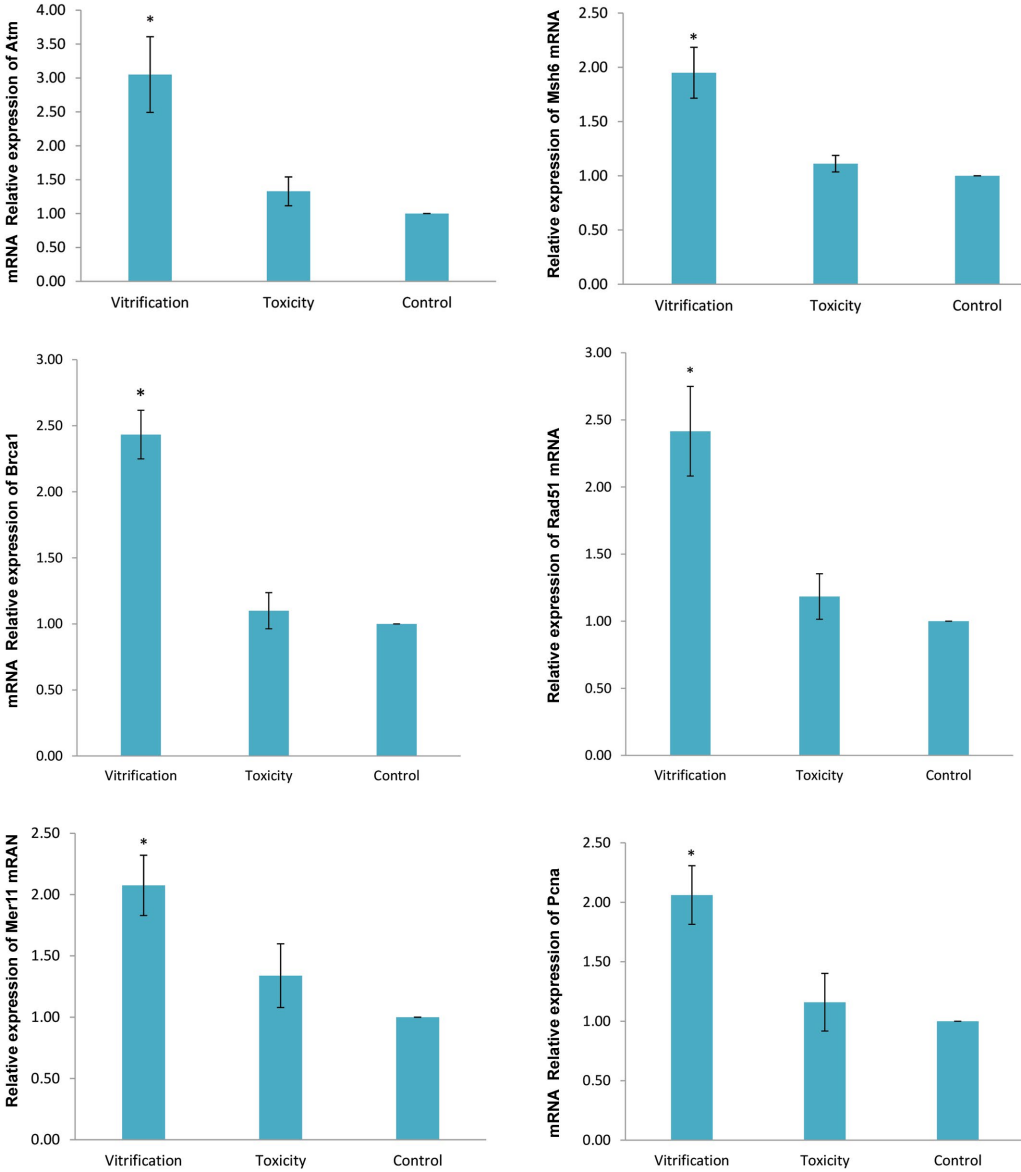
Relative mRNA expression levels of *Atr, Pcna, Brca1, Rad51, Msh6 *and *Mre11* in preantral follicles on the second day of cultivation period. Data are presented as mean ± SD. *; Indicates significant difference compared to the control (P<0.05).

**Fig.2:**
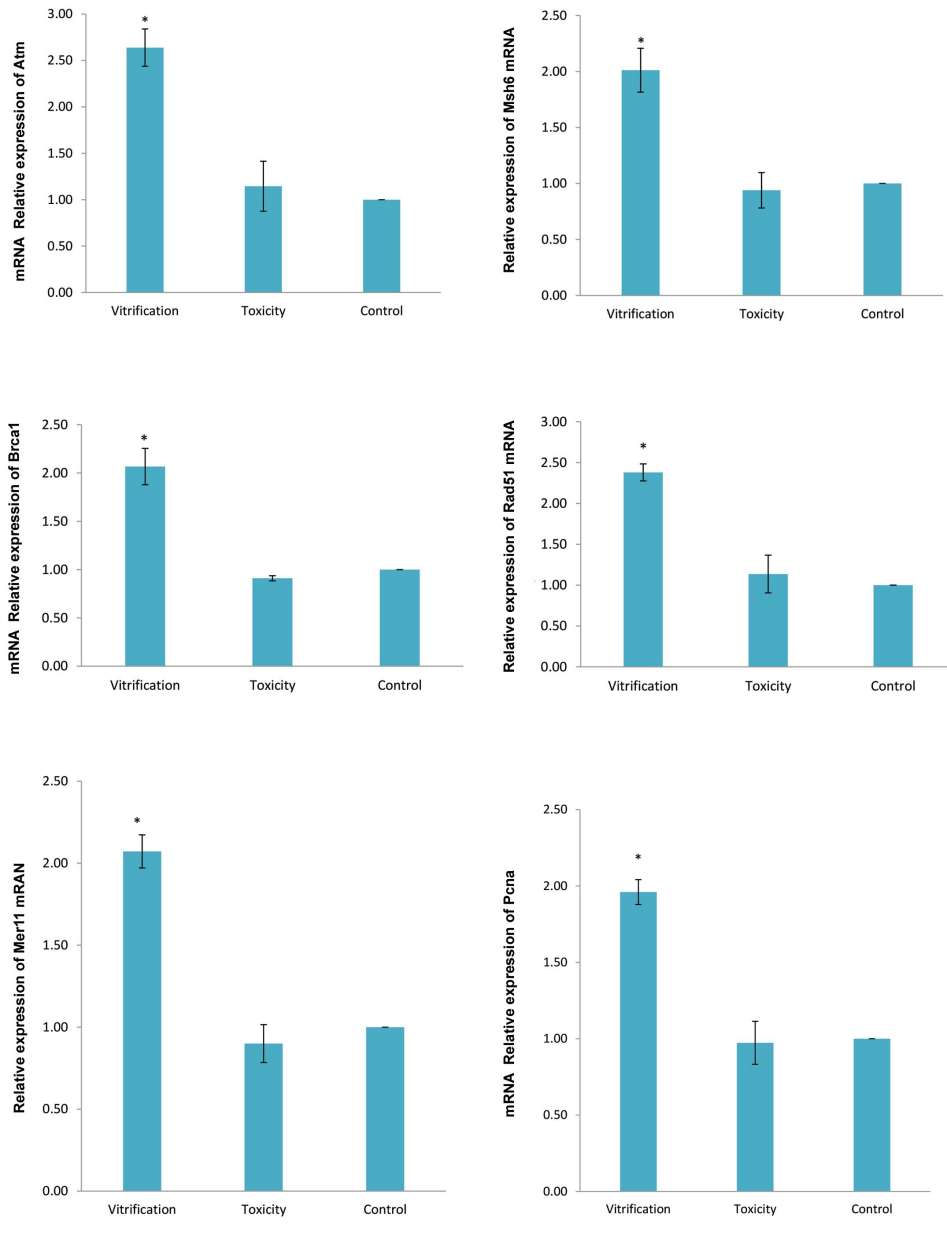
Relative mRNA expression levels of *Atr, Pcna, Brca1, Rad51, Msh6* and *Mre11* in preantral follicles on the sixth day of cultivation period. Data are presented as mean ± SD. *; Indicates significant difference compared to control (P<0.05).

Results of the statistical analysis of data for all three groups of control, vitrification and toxicity are presented in Figure 1 and Figure 2. Dunnett post hoc test showed that relative expression levels of *Atr, Pcna, Brca1, Rad51, Msh6* and *Mre11 *genes in the vitrification group was significantly higher than control and toxic groups, on the second and sixth days of culture period. While, the relative expression level of the aforementioned genes in the control and toxic groups was not significantly different.

## Discussion

Cryopreservation methods allow long-term preservation of any types of biological cells and tissues such as ovaries, ovarian follicles, oocytes, embryos and stem cells. While, various degrees of cell damage may occur during cryopreservation, which are depends on several factors such as size, shape, permeability, quality and sensitivity of the cell or tissue (17, 18). These damages in oocyte and embryo can include dispersion of cellular skeleton, releasing cortical granules, zona hardening and disruption of plasma membrane which in turn negatively affect developmental competence of oocyte and embryo (19). However, some effects of vitrification on the molecular structure, including DNA and mRNA expression of the genes, is not immediately expressed after warming and therefore does not necessarily cause cell degeneration, while they can disturb growth and development of the cell (3, 20-22).

The present study was aimed to investigate this issue and examine the effect of vitrification on the developmental competence of mouse ovarian preantral follicles and expression of some DNA repair genes. Results of the first step of the present study showed that the rates of growth, survival, antrum formation and ovulation in the vitrified group were significantly reduced compared to the control and toxicity groups. In agreement with these observations, other studies showed that growth and rate of developmental competence of preantral follicles isolated from the vitrified ovarian tissue is slower than the non-vitrified preantral follicles (9, 21). However, Mazoochi et al. showed that there was no significant difference between the rates of growth and survival of follicles obtained from vitrified ovaries and fresh ovaries (17, 18). The differences in the recent and other studies can be attributed to the different protocols and materials. One of the important factors in regulation of follicular development is growth factors secreted by granulosa cells (23). Choi et al. (24) observed that number of granulosa cells was decreased in the vitrified group compared to the fresh group. Whereas, concentration of growth factors did not change in vitrified follicles.

Since DNA repair pathways are important in genetic preservation of ovarian follicles, in the second step of present study, effect of vitrification on the expression pattern of some genes involved in DNA repair pathways of mouse preantral follicles were investigated. The results showed that relative expression of *Msh6, Pcna, Rad51, Brca1, Mre11* and *Atm* genes in the preantral follicles of the vitrified group were significantly increased in comparison with those of the control and toxicity groups on the second and sixth days of culture period. It seems that an increase in the expression of DNA repair genes is a response to the damages resulted from the vitrification on the DNA structure. In addition, similar relative expression of DNA repair gene in the control and toxicity groups might indicate that exposure to the cryoprotectants has no deleterious effect on the DNA structure.

Maintaining genomic integrity of the germ cells is vital role in cell cycles, because it allows the genetic information to be correctly transmitted to the next progeny (25, 26). It was demonstrated that DNA repair genes in the oocyte are more expressed than embryos, in response to DNA damage (12, 27). In this regard, Ménézo et al. (12) showed that DNA repair genes including *MSH6, BRCA1, MRE11* and *PCNA* were widely expressed in human GV oocytes. Moreover, it was established that the number of ovarian follicles in the mutated Brca1 mice was significantly reduced (13). During repair of DNA double strand breaks, a complex of the Brca1, Rad50, Mre11 and Atm proteins accompanied with forming Brca2 and Rad51 proteins complex to repair these lesions. If this repair does not occur, the cell eventually becomes apoptotic (10-12). Titus et al. showed that reduction in the expression of *Brca1* was associated with a significant decrease in the developmental competence of oocytes (13). *Other researchers investigated* the effect of cryopreservation on the gene expression involved in folliculogenesis (3, 28, 29). It was shown that cryopreservation decreased expression of *Fagl* gene, while increased expression of *Fshr* and *Gdf9* genes. This in turn confirmd that cryopreservation changed the gene expression patterns (30). Furthermore, in a study conducted by Ménézo et al. (12) effect of the slow freezing and vitrification on gene expression pattern of human MII oocytes were investigated and it was shown that pattern of gene expression was changed in the both methods. They showed that slow freezing increased expression of *BRCA1* gene. This finding is constant with the results of present study. While others found conflicting results, regarding the other genes (17, 18).

There may be some possible limitations in this study, including efficiency of the preantral follicle maturation and extruded oocyte maturation. One possible reason could be attributed to their culture condition *in vitro*. In the present study, there may be several differences between the follicle growth *in vivo* and follicle maturation *in vitro*. In the current *in vitro* culture system, intraovarian nutritional factors and growth factors improving follicles development through autocrine and paracrine manners were absent. However, supplementation of maturation medium with some of these factors may be efficient for follicle development. Another reason for the weakness of follicle growth rate in the present study may be resulted from culturing in the two-dimensional culture system. Three-dimensional culture system *in vitro* might be supportive to eliminate this problem. More investigations will be needed to verify this hypothesis. Another limitation of this study is the identification of growing follicles during dissecting from ovarian tissue. In the ovarian follicular development, a vital step is recruitment of follicles into growing cohort. Our culture system is potentially compatible with the conventional microscopically methods for assessment the follicles. Further criteria is essential for the identification of recruited follicles.

## Conclusion

The results of this study indicate that increasing expression of DNA repair genes can be a response of preantral follicles to repair DNA damages after vitrification.
